# Real-World Palbociclib Use in HR+/HER2− Advanced Breast Cancer in Canada: The IRIS Study

**DOI:** 10.3390/curroncol28010066

**Published:** 2021-01-24

**Authors:** Katie Mycock, Lin Zhan, Gavin Taylor-Stokes, Gary Milligan, Debanjali Mitra

**Affiliations:** 1 Adelphi Real World, Bollington SK10 5JB, UK; gavin.taylor-stokes@adelphigroup.com (G.T.-S.); gary.milligan@adelphigroup.com (G.M.); 2 Pfizer Inc., New York, NY 10017, USA; cheesezl@hotmail.com (L.Z.); Debanjali.Mitra@pfizer.com (D.M.)

**Keywords:** palbociclib, Canada, metastatic breast cancer, real-world, retrospective, medical chart review

## Abstract

Background: Palbociclib is a selective cyclin-dependent kinase (CDK) 4/6 inhibitor used in combination with aromatase inhibitors or fulvestrant for patients with hormone receptor-positive (HR+) human epidermal growth factor receptor 2 (HER2)-negative advanced/metastatic breast cancer (ABC/MBC). Palbociclib was the first CDK 4/6 inhibitor approved for HR+/HER2− ABC/MBC treatment in Canada in combination with letrozole (P+L) as an initial endocrine-based therapy (approved March 2016), or with fulvestrant (P+F) following disease progression after prior endocrine therapy (approved May 2017). The Ibrance Real World Insights (IRIS) study (NCT03159195) collected real-world outcomes data for palbociclib-treated patients in several countries, including Canada. Methods: This retrospective chart review included women with HR+/HER2− ABC/MBC receiving P+L or P+F in Canada. Physicians reviewed medical records for up to 14 patients, abstracting demographic and clinical characteristics, treatment patterns, and clinical outcomes. Progression-free rates (PFRs) and survival rates (SRs) at 6, 12, 18, and 24 months were estimated via Kaplan–Meier analysis. Results: Thirty-three physicians examined medical records for 247 patients (P+L, *n* = 214; P+F, *n* = 33). Median follow-up was 8.8 months for P+L and 7.0 months for P+F. Most patients were initiated on palbociclib 125 mg/d (P+L, 90.2%; P+F, 84.8%). Doses were reduced in 16.6% of P+L and 14.3% of P+F patients initiating palbociclib at 125 mg/d. The PFR for P+L was 90.3% at 12 months and 78.2% at 18 months; corresponding SRs were 95.6% and 93.0%. For P+F, 6-month PFR was 91.0%; 12-month SR was 100.0%. Conclusions: Dose reduction rates were low and PFR and SR were high in this Canadian real-world assessment of P+L and P+F treatments, suggesting that palbociclib combinations are well tolerated and effective.

## 1. Introduction

Breast cancer is predicted to be the most common non-melanoma cancer among women in Canada in 2020, with an estimated 27,400 breast cancer diagnoses and 5100 deaths [1]. Despite considerable advances in the diagnosis and treatment of breast cancer, the 5-year survival rate for patients with stage 4 disease has been estimated at 22% [2,3]; consequently, much remains to be done to improve outcomes for these patients.

Hormone receptor-positive, human epidermal growth factor receptor 2-negative (HR+/HER2–) breast cancer is the most common breast cancer subtype in Canada, occurring in 59−65% of women included in the Ontario Cancer Registry [4,5]. Endocrine agents have historically been the backbone of systemic treatment regimens for these cancers [6]; however, the complexity of breast cancer subtypes and their classification, as well as the emergence of endocrine resistance, have led to the development of targeted treatments to improve health outcomes in patients with advanced breast cancer (ABC) or metastatic breast cancer (MBC) [7]. Palbociclib (Ibrance: Pfizer, New York, NY, USA) was the first selective cyclin-dependent kinase (CDK) 4/6 inhibitor approved in Canada for use in combination with letrozole for the treatment of post-menopausal women with estrogen receptor-positive (ER+) HER2– ABC as an initial endocrine-based therapy for their metastatic disease [8], and later in combination with fulvestrant for the treatment of women with HR+/HER2– locally advanced or MBC whose disease progressed after prior endocrine therapy [9]. The approval of palbociclib was subsequently expanded to encompass overall use in combination with other aromatase inhibitors as initial endocrine-based therapy in post-menopausal women with HR+/HER2− ABC/MBC (June 2018) [10]. These approvals were based on the phase III PALOMA 2 and PALOMA 3 studies, respectively [11,12].

Although extensive clinical trial data are now available for palbociclib, evidence for its use in real-world patients with HR+/HER2− ABC/MBC is only just emerging. The Flatiron Health Analytic database has been used to compare the real-world efficacy of palbociclib + letrozole (*n* = 772) versus letrozole alone (*n* = 658) in the first-line setting [13]. In this retrospective analysis of patients with HR+/HER2– MBC treated with palbociclib + letrozole or letrozole monotherapy between February 2015 and February 2019, a significant overall survival (OS) and progression-free survival (PFS) benefit was observed for the combination versus letrozole alone. The median PFS for palbociclib + letrozole was 20.2 months (*p* < 0.0001 vs. letrozole alone after propensity score matching; hazard ratio = 0.54) over a median follow-up period of 24.4 months for patients in the palbociclib + letrozole group and 23.1 months for those in the letrozole group. Median OS was not reached (*p* < 0.0001 vs. letrozole alone after propensity score matching; hazard ratio = 0.58). The OS rate for palbociclib + letrozole was 80.1% at 24 months.

The multi-country Ibrance Real World Insights (IRIS) study (NCT03159195) aims to describe demographic, clinical characteristic, treatment, and outcomes data, including progression-free rates (PFRs) and survival rates (SRs), for patients who received palbociclib combinations in the real world setting. Upon completion, the IRIS study aims to have collected data for more than 2900 palbociclib-treated patients recruited by more than 400 physicians across 13 countries in North and South America, Europe, and Asia. The IRIS study aims to provide country-specific real-world data that can be used to complement clinical trial data with a larger and more diverse sample, thereby addressing the evidence gap between clinical trials and the real-world clinical setting. To date, results from the US, German, and Argentinian cohorts have been published, demonstrating favorable outcomes in palbociclib-treated patients in the real-world clinical setting in these countries [14,15,16]. We now report findings from the Canadian cohort of patients. 

## 2. Methods

### 2.1. Study Design

IRIS is a retrospective, physician-based, medical chart review of patients who received palbociclib in combination with either an aromatase inhibitor (e.g., letrozole) or fulvestrant according to labeled indications across multiple countries in North America, Europe, Latin America, and Asia. Data for the Canadian IRIS cohort of patients were collected between July and October 2019. At the time of data collection, treatment with palbociclib + an aromatase inhibitor was broadly available via provincial public reimbursement or private insurance; however, palbociclib + fulvestrant was only covered by private insurance. At the time of protocol development, palbociclib + letrozole was the only approved CDK 4/6 + aromatase inhibitor combination in Canada; other aromatase inhibitors that were subsequently approved are not reflected in the current study.

The study was conducted in accordance with the International Society for Pharmacoepidemiology recommendations and the Guidelines for Good Pharmacoepidemiology Practices. The study protocol was approved by the Western Institutional Review Board (20190576; 8 March 2019).

### 2.2. Study Population

Oncologists and hematologist-oncologists were included in the study if they had received their medical qualification >2 years but <35 years before the date of medical record abstraction and were treating at least four patients with HR+/HER2– ABC/MBC. Physicians had to be responsible for the initiation and management of the patient’s treatment.

Eligible patients were women aged ≥18 years with a diagnosis of HR+/HER2– ABC/MBC. Patients had received palbociclib combinations in accordance with labeled indications in Canada, i.e., palbociclib + letrozole as an initial endocrine-based therapy for post-menopausal women or palbociclib + fulvestrant for women with disease progression following endocrine therapy (pre- or peri-menopausal women were also receiving a luteinizing hormone-releasing hormone agonist).

To ensure sufficient follow-up for clinical outcomes data, patients had to have initiated palbociclib + letrozole ≥ 6 months or palbociclib + fulvestrant ≥ 3 months before the date of abstraction. Patients were selected sequentially, working forwards in time from the specified index date, which was defined as 60 days after the physician first prescribed palbociclib + letrozole or palbociclib + fulvestrant following regulatory approval of palbociclib in Canada. For example, palbociclib + letrozole was approved on 1 March 2016, and if the physician started prescribing this combination the next day, the index date would be 2 May 2016. No prior or current enrolment in interventional clinical trials for HR+/HER2− ABC/MBC was permitted.

### 2.3. Data Source and Extraction

Physicians abstracted data from medical records for up to 14 sequential patients meeting the inclusion criteria from the index date. Data were abstracted from the index date until the last available medical record, death, or date of record abstraction, whichever was earliest. Data were abstracted into online electronic case report forms. Abstracted data included demographic and clinical characteristics such as age, ethnicity/race, menopause onset status, Eastern Cooperative Oncology Group (ECOG) performance status (PS), and site(s) of metastases at MBC diagnosis. Treatment patterns captured included starting doses and modifications and reasons for palbociclib regimen changes and discontinuation. The clinical outcomes collected included best response to treatment and PFRs and SRs at various timepoints. PFRs beyond 6 months and SRs beyond 18 months for palbociclib + fulvestrant were immature at the time of analysis due to insufficient follow-up. Clinical benefit rates (CBRs) and objective response rates were also derived. Definitions of clinical response were provided to physicians. Definitions of all clinical outcome variables are presented in Table 1.

### 2.4. Statistical Analysis

Analyses were descriptive and no formal hypothesis was tested. Time-to-event outcomes were calculated using Kaplan–Meier estimates at 6-, 12-, 18-, and 24-month time points where available. CBR data were censored for patients with <24 weeks’ data who were still receiving palbociclib but had no evidence of complete response (CR), partial response (PR), or progressive disease. Missing data were not imputed. Analyses were conducted using STATA statistical software version 16.1 (StataCorp LLC, College Station, TX, USA).

### 2.5. Role of the Funding Organization

The funder of the study was involved in study design, data interpretation, and editing of the manuscript. Study design, data interpretation, data collection, data analysis, statistical analyses, and editing were done by Adelphi Real World, supported by the study funder. All authors had access to the aggregated data and had final responsibility for the decision to submit for publication.

## 3. Results

### 3.1. Physician and Patient Demographics

Between July and October 2019, 33 physicians (31 oncologists; 2 hematologist-oncologists) abstracted data for 247 patients; of these, 214 patients were treated with palbociclib + letrozole and 33 with palbociclib + fulvestrant. Nineteen physicians (57.6%) were from academic teaching hospitals, eleven (33.3%) were from cancer centers/clinics, and the remaining three (9.1%) were from community/non-teaching hospitals. Participating physicians represented Ontario (*n* = 9; 27.3%), Quebec (*n* = 8; 24.2%), Western Provinces (Manitoba and British Colombia; *n* = 11; 33.3%), and Atlantic Provinces (Nova Scotia and New Brunswick; *n* = 5; 15.2%). Patients were predominantly white/Caucasian (P+L: *n* = 153 (71.5%); P+F *n* = 25 (75.8%)), with a mean (±standard deviation (SD)) age of 61.9 (10.2) years (P+L: 61.9 (10.3) years; P+F: 62.3 (10.1) years).The majority of patients had an ECOG PS of 0/1 at palbociclib initiation (P+L: *n* = 175 (81.8%); P+F: *n* = 25 (75.8%)) and metastatic disease (P+L: *n* = 180 (84.1%); P+F: *n* = 24 (72.7%)); of those, over half of the patients had visceral disease (P+L: *n* = 109 (60.6%); P+F: *n* = 10 (41.7%)) (Table 2). The mean (±SD) follow-up durations for palbociclib + letrozole and palbociclib + fulvestrant were 11.0 (6.0) months and 7.1 (3.3) months, respectively (median: 8.8 months and 7.0 months, respectively) (Table 2).

### 3.2. Palbociclib + Letrozole

#### 3.2.1. Treatment Patterns 

The mean (±SD) time from diagnosis of HR+/HER2– ABC/MBC to initiation of palbociclib + letrozole was 2.9 (±8.9) months; 190 of 195 patients (97.4%) (195 with a known date of ABC/MBC diagnosis) initiated palbociclib treatment within 12 months of ABC/MBC diagnosis. Most patients (*n* = 205 (95.8%)) received palbociclib + letrozole as a first-line therapy in the advanced and metastatic setting. Although all patients received palbociclib + letrozole as initial endocrine-based therapy as per the labeled indication, a small proportion of patients received palbociclib + letrozole in the second line or later, following prior chemotherapy (second line *n* = 8 (3.7%); third line *n* = 1 (0.5%)).

The most common starting dose of palbociclib + letrozole was 125 mg/d (*n* = 193 (90.2%)) (Table 3). The most common reasons for initiating treatment at a dose of <125 mg/d were avoidance of toxicity (*n* = 13 (61.9%)), patient age (*n* = 7 (33.3%)), and ECOG PS (*n* = 5 (23.8%)). More patients with an ECOG PS ≥2 initiated at a lower dose (*n* = 6 of 39 patients (15.4%) initiated at 100 mg/d) than those with ECOG PS of 0/1 (PS 0: *n* = 3 of 83 (3.6%); PS 1: *n* = 12 of 92 (13.0%) initiated at ≤100 mg/d). More patients aged ≥65 years started on a dose of ≤100 mg/d (*n* = 15 of 97 (15.5%)) than those <65 years (*n* = 6 of 117 (5.1%)).

Regardless of starting dose, dose adjustments occurred in 42 patients (19.6%): dose reductions were experienced by 33 patients (15.4%), 5 (2.3%) had cycle delay(s), 3 (1.4%) had dose increase(s), and 2 (0.9%) had dose interruption(s). Of those experiencing a dose adjustment, ten (23.8%) patients experienced more than one dose change. All dose reductions were a result of side effects/toxicity (*n* = 33 (100.0%)). Among the 193 patients who started on a palbociclib dose of 125 mg/d, 32 (16.6%) required a dose reduction.

Palbociclib treatment was ongoing in 186 patients (86.9%) at the time of data extraction. Among the 28 patients (13.1%) who had discontinued treatment, 27 had a reason recorded by their physician for doing so: twelve patients (44.4%) had disease progression following initial disease control/response and eight patients (29.6%) had disease progression without initial disease control/response.

#### 3.2.2. Clinical Outcomes

**Figure 1:**
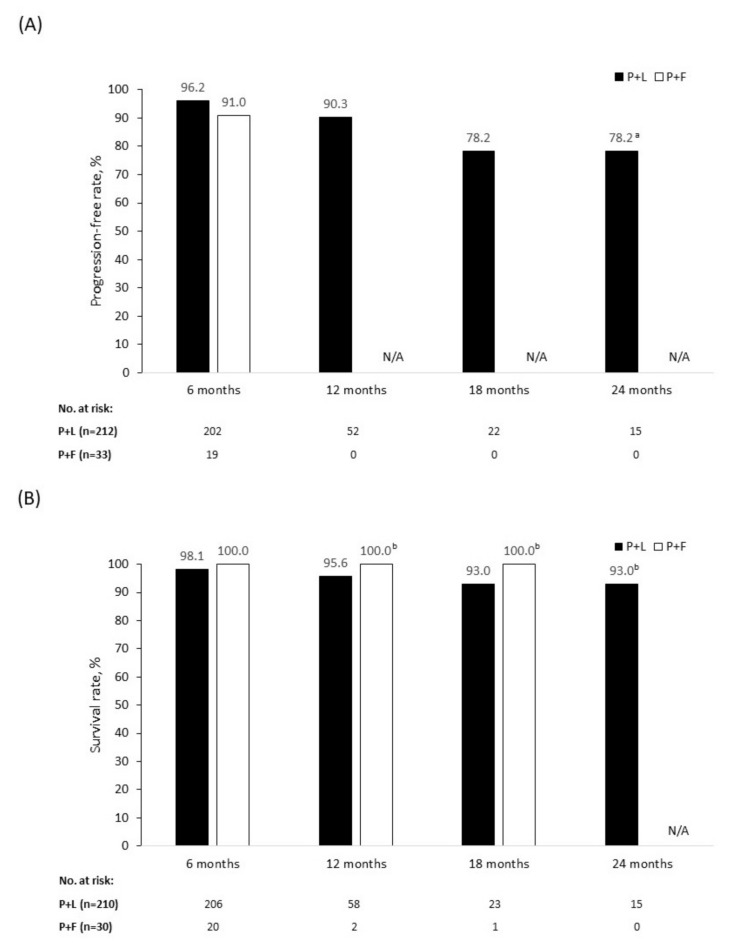
Progression-free rate (**A**) and survival rate (**B**) in patients treated with palbociclib + letrozole (P+L) and palbociclib + fulvestrant (P+F). N/A = not applicable. ^a^ Censored data from patients who remained on treatment in whom a progression event did not occur during the 18–24-month time period. ^b^ Censored data from patients who remained on treatment in whom a death event did not occur during the follow-up period. P+F: No 12-, 18-, and 24-month progression-free or 24-month survival data available because of the insufficient follow-up period. For patients who remained on treatment, no death events occurred during the follow-up period. P+L: For patients who remained on treatment, no progression or death events occurred during the 18–24-month follow-up period.

As their best response, 22 (10.3%) patients achieved a CR and 151 (70.6%) patients achieved a PR. The mean (±SD) time to CR after palbociclib initiation was 4.7 (±3.0) months and the mean (±SD) time to PR was 3.4 (±1.3) months. Overall, 173 patients (80.8%) had an objective response to treatment and 201 (93.9%) had achieved a clinical benefit (Table 4). During the follow-up period, 20 patients (9.3%) had disease progression. Estimated by Kaplan–Meier analysis, the 12- and 18-month PFRs were 90.3% and 78.2%, respectively (Figure 1A). At the time of data collection, nine patients (4.2%) had died; 95.6% patients were alive at 12 months and 93.0% at 18 months (Figure 1B).

### 3.3. Palbociclib + Fulvestrant

#### 3.3.1. Treatment Patterns

Palbociclib + fulvestrant was prescribed as the first-line treatment for 12 patients (36.4%) and as a second-line or later treatment for 21 patients (63.6%). The mean (±SD) time from diagnosis of HR+/HER2– ABC/MBC to palbociclib + fulvestrant initiation was 9.5 (±13.7) months. The most common prior therapies in the advanced setting were endocrine therapy (*n* = 20 (95.2%)), targeted therapy (*n* = 2 (9.5%)), and chemotherapy (*n* = 2 (9.5%)) (Table 2). 

The most common starting dose was 125 mg/d (*n* = 28 (84.8%)) (Table 3). Among the five patients who initiated palbociclib at a dose of <125 mg/d, the most common reasons were avoidance of toxicity (*n* = 5 (100.0%)), patient age (*n* = 3 (60.0%)), and ECOG PS (*n* = 2 (40.0%)). 

Overall, dose adjustments occurred in four patients (12.1%); all four patients (100.0%) had one or more dose reduction and one patient (25.0%) also had dose interruption(s). All dose reductions were due to side effects/toxicity. Among the 28 patients who started on a palbociclib dose of 125 mg/d, four patients (14.3%) had a dose reduction.

At the time of data abstraction, palbociclib treatment was ongoing in 30 patients (90.9%). Three patients (9.1%) discontinued treatment, two (66.7%) because of disease progression following initial control/response and one (33.3%) because of disease progression with no initial control/response.

#### 3.3.2. Clinical Outcomes

**Figure 2:**
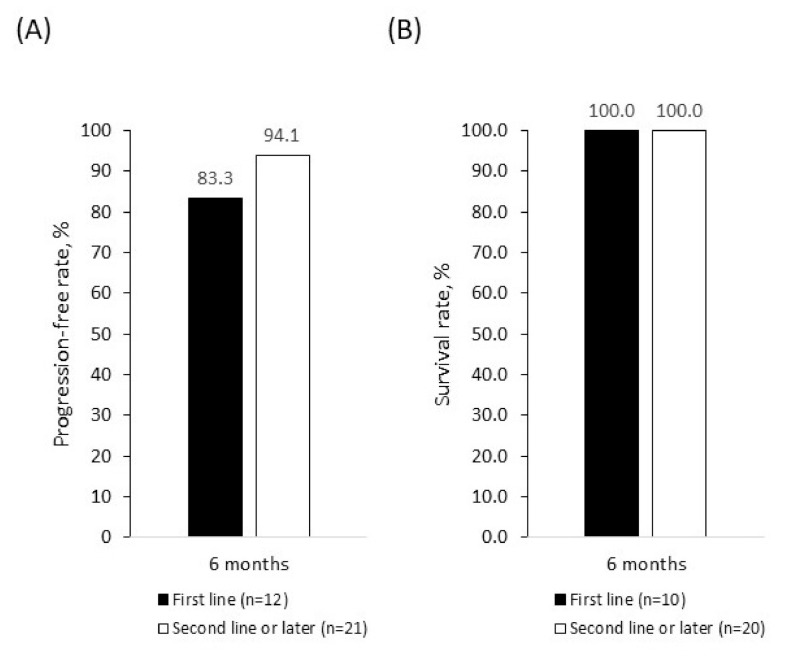
Progression-free rate (**A**) and survival rate (**B**) in patients treated with palbociclib + fulvestrant as first-line or second- and later-line therapy. Rates were estimated using Kaplan–Meier analyses.

As a best response, four patients (12.5%) achieved a CR and 20 patients (62.5%) achieved a PR. The mean (±SD) time to CR after palbociclib initiation was 4.0 (±1.4) months and time to PR was 2.9 (±0.9) months. Overall, 24 patients (75.0%) had an objective response to treatment and 30 (93.8%) achieved clinical benefit (Table 4). Three patients (9.1%) had disease progression by the time of data collection. The 6-month PFR was 91.0% overall (Figure 1A), 83.3% in patients receiving first-line palbociclib + fulvestrant, and 94.1% in those receiving palbociclib + fulvestrant in the second line or later (Figure 2A). There were no deaths among patients treated with palbociclib + fulvestrant at the time of data collection, giving an SR of 100.0% across all lines of therapy (Figure 1B and B) during the follow-up period. Because of the limited time on treatment and the small patient population analyzed, PFR beyond 6 months and SR beyond 18 months were not available. 

## 4. Discussion

Designed to address the lack of real-world data for palbociclib globally, the IRIS study investigated treatment patterns and clinical outcomes in patients with HR+/HER2– ABC/MBC who were treated with palbociclib in multiple countries in North America, Latin America, Europe, and Asia.

Canadian real-world treatment patterns were consistent with previous reports in the US, Argentina, and Germany [14,15,16]. The most commonly prescribed palbociclib dose was 125 mg/d, in line with the prescribing information [10], although lower doses were used at initiation in some patients, primarily to avoid toxicity. The 125 mg/d dose appeared to be well tolerated by patients in Canada, with 83.7% of patients remaining at this dose. Among patients initiating palbociclib on 125 mg/day, only 16.6% in the palbociclib + letrozole group had a dose reduction, while 14.3% of patients in the palbociclib + fulvestrant group had their dose reduced, all to avoid side effects/toxicity. Palbociclib dose reductions appear to be less frequent in these settings than previously reported in clinical trials. The palbociclib dose was reduced in 36.0% of patients treated with palbociclib + letrozole in PALOMA-2 [17] and in 33.9% of patients treated with palbociclib + fulvestrant in PALOMA-3 [18]. However, the PALOMA studies mandated dose initiation at 125 mg/day, whereas in real-world clinical practice, patients may be initiated at lower doses. Differences between the IRIS and PALOMA findings are likely multifactorial and related to study designs and patient populations. Unlike the strictly controlled PALOMA study populations, IRIS included patients with ECOG PS >2, perimenopausal women (in the palbociclib + letrozole group), no limit regarding concomitant medication or patient comorbidities, and no requirements for adequate organ function. Furthermore, clinical endpoints in IRIS did not require confirmation via imaging according to Response Evaluation Criteria in Solid Tumors (RECIST).

Regardless of starting dose, palbociclib doses were reduced in 15.4% of patients receiving palbociclib + letrozole and in 12.1% of those receiving palbociclib + fulvestrant. These Canadian data are in line with observations from the IRIS US cohort, in which 15.5% of palbociclib + aromatase inhibitor and 11.0% of palbociclib + fulvestrant patients had a dose reduction, and from the German cohort, where 14.5% of palbociclib + aromatase inhibitor and 11.1% palbociclib + fulvestrant patients had a dose reduction [15,16]. Rates of dose reduction were similar in the IRIS Argentina cohort, where 16.2% of palbociclib + letrozole patients and 5.3% palbociclib + fulvestrant patients experienced a dose reduction; however, the palbociclib + fulvestrant cohort was much smaller in Argentina [14]. 

Patients receiving palbociclib in the Canadian IRIS cohort had favorable response rates, PFRs, and SRs. A total of 78.2% of patients receiving palbociclib + letrozole remained progression-free after 18 months, as estimated by the Kaplan–Meier calculation, and 91.0% of patients receiving palbociclib + fulvestrant were progression-free after 6 months. Furthermore, the SR at 18 months was 93.0% for patients receiving palbociclib + letrozole and 100.0% for those receiving palbociclib + fulvestrant. Although differences in patient characteristics and assessment methods between IRIS and the PALOMA studies limit the degree to which the outcomes of these studies can be compared, response and survival data from IRIS complement the efficacy data for palbociclib combinations observed in the PALOMA studies [11,18].

Some limitations of this study warrant consideration. As with studies of this nature, only physicians willing to participate were included, which may result in potential selection bias. To minimize this, recruitment of physicians across all geographic regions and treatment settings was ensured. Additionally, to mitigate any patient selection bias, physicians were asked to select consecutive patients in line with the index date. The palbociclib + fulvestrant sample was relatively small (*n* = 33), which may be related to palbociclib + fulvestrant combinations only being available via private insurance at the time of data collection. Because of the short follow-up time available, caution must be exercised when interpreting outcomes data in this group; progression and survival data were not available at 12 months and beyond for patients in the palbociclib + fulvestrant group. Finally, in this study, letrozole was specified as the aromatase inhibitor partner for palbociclib as this was the approved combination at the time of protocol development. Although palbociclib has since been approved and reimbursed in combination with other aromatase inhibitors, other combinations are not reflected in this study.

## 5. Conclusions

In conclusion, the IRIS study provides novel information regarding real-world treatment patterns and clinical outcomes associated with palbociclib in combination with letrozole or fulvestrant for patients with ABC/MBC in Canada, supplementing data previously reported from clinical studies. The efficacy of palbociclib combinations was favorable, as reflected by PFRs and SRs. Dose-reduction rates were low, suggesting that these combinations are well tolerated in the real-world setting. Ongoing studies are required to deliver mature outcome data beyond 12/24 months in this setting.

## Figures and Tables

**Table 1:** Clinical outcome definitions.

Clinical Outcome	Definition/Variables
**Clinical response**	
Complete response	Where “complete response” has been recorded at any time (no 24-week minimum).
Partial response	Where “partial response” has been recorded at any time (no 24-week minimum).
Stable disease ≥24 weeks	Patient remained on palbociclib for a minimum of 24 weeks, without complete or partial response, death, treatment switch, or progression.
Stable disease <24 weeks	Stable disease recorded for initial response, with a subsequent progression recorded within <24 weeks or treatment switch for reason other than progression within <24 weeks or death without recorded progression within <24 weeks.
Progressive disease	Progressive disease recorded for initial response without a subsequent partial or complete response recorded.
**Derived clinical endpoints**	
Objective response rate	Proportion of patients achieving a complete or partial response as assessed by the physician and reported in the patient records; radiologic confirmation was not required and no criteria to re-evaluate were provided.
Clinical benefit rate	Proportion of patients who achieved a complete or partial response or had stable disease for ≥24 weeks as assessed by the physician.
Progression-free rate	Proportion of patients with no evidence of progression or death at 6, 12, 18, and 24 months.
Survival rate	Proportion of patients alive at 6, 12, 18, and 24 months.

**Table 2:** Patient demographic, clinical, and treatment characteristics.

Characteristic	Overall (*n* = 247)	P+L (*n* = 214)	P+F (*n* = 33)
**Age at palbociclib initiation, years**			
Mean (SD)	61.9 (10.2)	61.9 (10.3)	62.3 (10.1)
Median (range)	62.0 (36.0–88.0)	63.0 (36.0–88.0)	60.0 (41.0–85.0)
<65, *n* (%)	139 (56.3)	117 (54.7)	22 (66.7)
≥65, *n* (%)	108 (43.7)	97 (45.3)	11 (33.3)
**Ethnicity, *n* (%)**			
White/Caucasian	178 (72.1)	153 (71.5)	25 (75.8)
Asian	34 (13.8)	29 (13.6)	5 (15.2)
Middle Eastern	11 (4.5)	10 (4.7)	1 (3.0)
Other	24 (9.7)	22 (10.3)	2 (6.1)
**Menopause status, *n* (%)**			
Natural menopause	224 (90.7)	193 (90.2)	31 (93.9)
Menopause induced by surgery	6 (2.4)	5 (2.3)	1 (3.0)
Menopause induced by LHRH suppression	17 (6.9)	16 (7.5)	1 (3.0)
**ECOG PS at palbociclib initiation, *n* (%)**			
0	92 (37.2)	83 (38.8)	9 (27.3)
1	108 (43.7)	92 (43.0)	16 (48.5)
2	42 (17.0)	36 (16.8)	6 (18.2)
3	5 (2.0)	3 (1.4)	2 (6.1)
**Stage at ABC/MBC diagnosis, *n* (%)**			
Locoregionally advanced (IIIb, IIIc)	43 (17.4)	34 (15.9)	9 (27.3)
Metastatic (stage IV)	204 (82.6)	180 (84.1)	24 (72.7)
**Occurrence of breast cancer, *n* (%)**			
Recurrent	89 (36.0)	61 (28.5)	28 (84.8)
De novo	158 (64.0)	153 (71.5)	5 (15.2)
**Metastatic sites, *n* (%)**			
No. of patients	204	180	24
Bone	136 (66.7)	119 (66.1)	17 (70.8)
Lung	81 (39.7)	75 (41.7)	6 (25.0)
Lymph nodes	62 (30.4)	55 (30.6)	7 (29.2)
Liver	43 (21.1)	38 (21.1)	5 (20.8)
Other	31 (15.2)	28 (15.6)	3 (12.5)
Visceral disease	119 (58.3)	109 (60.6)	10 (41.7)
Non-visceral disease	85 (41.7)	71 (39.4)	14 (58.3)
**Prior therapy for ABC/MBC, *n* (%)**			
No. of patients	30	9	21
Endocrine therapy ^a^	20 (66.7)	0 (0.0)	20 (95.2)
Chemotherapy	11 (36.7)	9 (100.0)	2 (9.5)
Targeted therapy ^a^	2 (6.7)	0 (0.0)	2 (9.5)
**Lines of treatment for ABC/MBC, *n* (%)**			
1	204 (82.6)	193 (90.2)	11 (33.3)
2	34 (13.8)	15 (7.0)	19 (57.6)
3	8 (3.2)	5 (2.3)	3 (9.1)
4	1 (0.4)	1 (0.5)	0 (0.0)

ABC = advanced breast cancer; ECOG PS = Eastern Cooperative Oncology Group performance status; LHRH = luteinizing hormone-releasing hormone; MBC = metastatic breast cancer; P+F = palbociclib + fulvestrant; P+L = palbociclib + letrozole; SD = standard deviation. ^a^ Targeted and endocrine therapies were not an option before palbociclib + letrozole approval in Canada.

**Table 3:** Doses and adjustments in patients treated with P+L or P+F.

Dose	P+L (*n* = 214)	P+F (*n* = 33)
**Starting dose, *n* (%)**		
125 mg/d	193 (90.2)	28 (84.8)
100 mg/d	17 (7.9)	4 (12.1)
75 mg/d	4 (1.9)	1 (3.0)
**Reason for starting dose <125 mg/d, *n* (%) ^a^**		
No. of patients	21	5
To avoid toxicity	13 (61.9)	5 (100.0)
Age	7 (33.3)	3 (60.0)
ECOG PS	5 (23.8)	2 (40.0)
Presence of comorbidities	4 (19.0)	0 (0.0)
**Dose adjustment, *n* (%)**		
Dose reduction	33 (15.4)	4 (12.1)
Dose increase	3 (1.4)	0 (0.0)
Dose interruption	2 (0.9)	1 (3.0)
Cycle delay	5 (2.3)	0 (0.0)
**Treatment status**		
Treatment ongoing	186 (86.9)	30 (90.9)
Treatment discontinued	28 (13.1)	3 (9.1)
**Reason for discontinuation, n (%)** **b**		
No. of patients	27	3
PD following initial control/response	12 (44.4)	2 (66.7)
PD without initial control/response	8 (29.6)	1 (33.3)
Patient request	3 (11.1)	0 (0.0)

ECOG PS = Eastern Cooperative Oncology Group performance status; PD = progressive disease; P+F = palbociclib + fulvestrant; P+L = palbociclib + letrozole. ^a^ Most common responses (≥15% overall); other reasons included “Due to line of therapy received” (P+L: *n* = 2 (9.5%); P+F: *n* = 1 (20.0%)), “patient request” (P+L: *n* = 2 (9.5%); P+F: *n* = 1 (20.0%)), and “concomitant medications” (P+L: *n* = 2 (9.5%)). Physicians could select multiple reasons for reduced starting dose. ^b^ Most common responses (≥10%); others included “treatment cost” (P+L: *n* = 1 (3.7%)), “side effects/toxicity” (P+L: *n* = 1 (3.7%)), “other” (P+L: *n* = 1 (3.7%)), and “don’t know” (P+L: *n* = 2 (7.4%)). Physicians could select multiple reasons for discontinuation.

**Table 4:** Outcomes in patients treated with palbociclib combination therapy.

			P+F		
Outcome	Overall (*n* = 247)	P+L ^a^ (*n* = 214)	All (*n* = 33)	First Line (*n* = 12)	Second Line or Line (*n* = 21)
**Best response, *n* (%)**					
No. of patients	246	214	32	11	21
Complete response	26 (10.6)	22 (10.3)	4 (12.5)	1 (9.1)	3 (14.3)
Partial response	171 (69.5)	151 (70.6)	20 (62.5)	6 (54.5)	14 (66.7)
Stable disease ≥24 weeks	30 (12.2)	28 (13.1)	2 (6.3)	1 (9.1)	1 (4.8)
Stable disease <24 weeks	3 (1.2)	1 (0.5)	2 (6.3)	1 (9.1)	1 (4.8)
Progressive disease	12 (4.9)	12 (5.6)	0 (0.0)	0 (0.0)	0 (0.0)
**Objective response rate, *n* (%)**	197 (80.1)	173 (80.8)	24 (75.0)	7 (63.6)	17 (81.0)
**Clinical benefit rate, *n* (%) ^b^**					
Upper bound	231 (93.9)	201 (93.9)	30 (93.8)	10 (90.9)	20 (95.2)
Lower bound	227 (92.3)	201 (93.9)	26 (81.3)	8 (72.7)	18 (85.7)
**Progression-free rate, % ^c^**					
No. of patients	245	212	33	12	21
6 months	95.8	96.2	91.0	83.3	94.1
12 months	88.7	90.3	–	–	–
18 months	76.9	78.2	–	–	–
24 months	76.9	78.2	–	–	–
**Survival rate, % ^d^**					
No. of patients	240	210	30	10	20
6 months	98.3	98.1	100.0	100.0	100.0
12 months	95.9	95.6	100.0	100.0	–
18 months	93.4	93.0	100.0	100.0	–
24 months	93.4	93.0	–	–	–

P+F = palbociclib + fulvestrant; P+L = palbociclib + letrozole. See Table 1 for response definitions. ^a^ Patients receiving P+L as a first-line endocrine-based therapy (*n* = 9 patients received P+L in the second line or as later advanced therapy, following prior chemotherapy). ^b^ Upper bound includes stable disease censored; lower bound excludes stable disease censored. Data were censored for clinical benefit rate if a patient was still receiving palbociclib but had <24 weeks’ data available and no evidence of complete or partial response or progressive disease. ^c^ Proportion of patients with no evidence of progressive disease or death. ^d^ Proportion of patients alive.
